# Building Parental Trust in Childhood Vaccination: Lessons From Iran’s COVID-19 Response

**DOI:** 10.34172/ijhpm.8960

**Published:** 2025-11-05

**Authors:** Shahin Mafinezhad, Zainab Alimoradi, Hasan Namdar Ahmadabad

**Affiliations:** ^1^Department of Pediatrics, School of Medicine, North Khorasan University of Medical Sciences, Bojnurd, Iran.; ^2^Social Determinants of Health Research Center, Research Institute for Prevention of Non-Communicable Diseases, Qazvin University of Medical Sciences, Qazvin, Iran.; ^3^Vector-Borne Diseases Research Center, North Khorasan University of Medical Sciences, Bojnurd, Iran.

 Since the 18th century, when Edward Jenner introduced vaccination to control smallpox, humanity has mitigated numerous pandemics through this method.^1^ Vaccines, comprising pathogen components or weakened/inactivated forms, stimulate the immune system to generate memory for future pathogen responses. Pandemic experiences underscore that safe, effective, affordable vaccines with equitable distribution are vital for societal health and well-being.^2^

 Achieving herd immunity to halt infectious disease spread requires policy-makers to provide vaccines cost-effectively and equitably while fostering public trust and acceptance. Factors like social, economic, cultural, religious influences, misinformation, and health literacy impact vaccine uptake.^3^ Vaccinating all, including children, is crucial; it prevents child hospitalizations and deaths, reduces social isolation and educational disruptions, and curbs transmission to vulnerable individuals.^4^

 COVID-19 emerged in Iran with confirmed cases reported on February 19, 2020, in Qom, though evidence indicates an earlier start in January 2020. By August 2020, official figures exceeded 350 000 cases and 20 000 deaths. As of 2022, cumulative cases reached over 7.2 million with approximately 141 000 deaths.^5^

 Iran faced multifaceted challenges in managing the COVID-19 pandemic, exacerbated by international sanctions that impeded imports of essential medical supplies, leading to shortages and heightened mortality.^6^ Governance flaws in the National Headquarters Against COVID-19, including poor coordination and contradictory policies, eroded public trust and compliance, with adherence dropping below 40%. Health system strains involved inadequate physical structures, human resource shortages, fatigue, and financial constraints, necessitating inter-sectoral reforms.^7^ Additionally, influential clerics sometimes propagated misinformation and opposed quarantines, hindering prevention efforts despite their potential as social assets.^8^

 Iran’s COVID-19 vaccination began in February 2021, with 155 million doses administered by year-end using inactivated (eg, Sinopharm), adenoviral (eg, Sputnik V), and recombinant platforms mortality.^5^ Despite challenges—including initial delays, distrust of Western vaccines, slow procurement, US/UK vaccine prohibitions, economic sanctions, lack of confidence in Chinese/Russian vaccines, financial/resource shortages for procurement, and inadequate production due to insufficient scientific/economic infrastructure^9^—coverage reached 75% for one dose and 65% fully vaccinated, reducing hospitalizations and mortality.^5^

 In the United States, three COVID-19 vaccines are available for children: Pfizer-BioNTech, Moderna, and Novavax. However, as a developing country, Iran could not access these and instead used the indigenous PastoCovac and Chinese Sinopharm vaccines for children aged 5-12 years. COVID-19 vaccine uptake among Iranian children was low (29%-60%). Parental hesitancy mainly arises from concerns about side effects, potential effects on growth and fertility, and perceived ineffectiveness (50.9% doubt protection).^10^ Other barriers include beliefs that the pandemic has ended, insufficient information, overcrowded vaccination sites, limited vaccine availability, and distrust in primary health care.^11,12^ Our study in Bojnourd, Iran, found over 60% of parents of children aged 5–12 years unwilling to vaccinate due to health system distrust, misinformation, and vaccine safety fears.^13^ Younger children, boys, those with pre-existing conditions, lower socioeconomic status, and children of hesitant mothers are less likely vaccinated, while older children, those with prior COVID-19 infection, and higher socioeconomic families show greater acceptance.^14^ In a systematic review and meta-analysis, we assessed global parental acceptance of COVID-19 vaccination for children, revealing a low rate of 57%. Influencing factors included country income level, WHO region, vaccine side effects, efficacy and benefit doubts, cost, accessibility, and trust in government and health systems.^15^

 A primary reason for parental hesitancy toward pediatric COVID-19 vaccination in Iran is uncertainty about vaccine safety and fear of adverse effects; over 81% of hesitant or unwilling parents in a Tabriz study cited side effect concerns.^11^ An Iranian study of PastoCovac and Sinopharm vaccines in children aged 5-12 years reported side effect incidences of 24%-37%, varying by type and dose. Most were localized and mild, resolving within days without treatment; no severe effects occurred.^16^

 Drawing upon findings from our previous studies and Iran’s unique COVID-19 experiences, we propose evidence-based policy recommendations to enhance parental acceptance of childhood vaccination in future pandemics:

- Improving public trust in health systems is essential for increasing parents’ willingness to vaccinate children, as studies during the COVID-19 pandemic linked low trust to vaccine hesitancy. For example, in Israel, strong trust in physicians promoted compliance among young, urban, educated parents, while involving community leaders improved adherence in minority groups.^17^ In Australia, vague government messaging intensified hesitancy, but transparent information and community partnerships restored trust and boosted routine adolescent vaccine uptake.^18^
- To increase parental acceptance of childhood vaccinations, public communication should use evidence-based social marketing, as shown in South Korea’s COVID-19 campaign, which achieved 94.8% second-dose coverage through proactive, credible messaging that countered misinformation, emphasized social norms, and maintained coherence.^19^ Additionally, building trust in healthcare providers, public health agencies, and pharmaceutical companies is essential. Communications should clearly highlight vaccine production, components, safety, and effectiveness with a positive tone to encourage rational evaluation and hope, boosting uptake among the public and health professionals.^20^
- To boost parental acceptance of childhood vaccinations via diverse options, health policy-makers must support researchers and investors in indigenous production technologies while enhancing distribution and supply chains. Africa’s experience highlights this urgency: despite consuming 25% of global vaccines, only 1% are manufactured locally across 11 facilities, with enablers like technology transfers, regulatory enhancements, and GAVI’s $1 billion accelerator targeting priority vaccines such as measles-rubella and yellow fever.^21^ Joint ventures with reputable international firms can accelerate tacit knowledge sharing, build regional hubs, secure varied imports during pandemics, and mitigate funding challenges.^22^
- The health system should prioritize pediatric vaccine research across diverse centers, focusing on safety and efficacy, as highlighted by the need for rapid, iterative studies post-COVID-19. Findings should be published in peer-reviewed journals and shared in accessible media formats. Strategic social media use, employs evidence-based communication and direct misinformation refutation to engage hesitant parents. Also tailored knowledge translation tools and effective social media strategies^23^ boost vaccine acceptance.
- To increase parental trust in pediatric vaccines, transparent communication of clear, accurate information about vaccines approved by trusted organizations like the World Health Organization (WHO), European Medicines Agency, Food and Drug Administration, Paul-Ehrlich-Institut, and Therapeutic Goods Administration is essential. Rigorous clinical trials and global pharmacovigilance ensure safety and efficacy for children. International safety monitoring collaborations quickly address concerns, further building trust.^24^ Clear, open communication of these processes effectively reduces vaccine hesitancy.
- Healthcare professionals, including pediatricians, family physicians, and school teachers, are key to increasing parental vaccine awareness. Collaborating with these groups builds trust and encourages childhood vaccination. For instance, well-informed physicians reduced COVID-19 vaccine hesitancy by 71%.^25^ School-based programs sustained positive vaccine attitudes despite access challenges.^26^
- Addressing vaccine hesitancy rooted in religious beliefs requires strategic engagement with religious leaders to influence their communities. Tailored messaging on vaccine necessity and safety counters misinformation. For example, leaders’ endorsements boosted pediatric COVID-19 vaccine acceptance by 19% in faith-based groups.^27^ This communication should emphasize the necessity and benefits of vaccinating children and dispel false beliefs and misinformation about vaccines.
- Evidence shows that addressing parental psychological barriers to childhood vaccine acceptance is complex.^28^ Key factors include cognitive attitudes toward vaccine safety and efficacy, trust in institutions, social norms, perceived behavioral control, peer influence, and personal factors like anxiety and pandemic-related disruptions. Effective strategies extend beyond scientific evidence, requiring demonstrated institutional competence, simplified logistics, clear risk-benefit communication, and leveraging widespread acceptance to promote positive norms.
- An online system like the US Vaccine Adverse Event Reporting System is essential for monitoring children’s vaccine side effects, enabling post-marketing surveillance. A 31-year Vaccine Adverse Event Reporting System evaluation (1990-2021) analyzed 1.74 million reports, with 2021 COVID-19 peaks at 48.52% domestic, influenced by regulations.^29^ Healthcare workers must record effects, sharing periodic reports with committees, institutions, and the public.
- Drawing from COVID-19 experiences, health policy-makers should partner with non-governmental organizations, such as Macao’s civil organizations, to educate parents via social media on vaccine importance, effectiveness, and safety while addressing hesitancy and facilitating access. For instance, Macao’s government-non-governmental organization partnerships shaped public agendas, boosted engagement, and achieved 85.6% two-dose population coverage by June 2022.^30^ Such collaborations can enhance parental acceptance of children’s vaccines for future pandemics.

## Acknowledgements

 We gratefully acknowledge the contributions of all authors whose articles informed and enriched this manuscript.

## Ethical issues

 Since the data were extracted from a public database, there was not necessary to get an ethics approval.

## Conflicts of interest

 Authors declare that they have no conflicts of interest.

## Declaration of Generative Artificial Intelligence (AI)

 While preparing this work, the authors used the GPT-4o service developed by OpenAI to improve the English language and remove grammar and spelling errors. After using this service, the authors reviewed and edited the content if needed and took full responsibility for the publication’s content.
